# The greatest challenge in contemplative science: tailoring meditative practices

**DOI:** 10.3389/fpsyg.2024.1483342

**Published:** 2024-12-17

**Authors:** Javier Garcia-Campayo, Roberto Whyte, Rinchen Hijar-Aguinaga

**Affiliations:** ^1^Department of Psychiatry, University of Zaragoza, Zaragoza, Spain; ^2^Department of Psychiatry, Enneagram Teacher, Madrid, Spain

**Keywords:** meditation, Buddhism, tailoring, personality, enneagram

## Abstract

The practice of meditation has grown in popularity around the world in recent decades. However, standard and untailored meditative practices are recommended for all practitioners, regardless of their personality traits or characteristics. No scientific attempts have been made to match specific meditation types with personality. This paper summarizes the limited proposals relevant to this subject in contemplative traditions such as Buddhism and schools of humanistic and transpersonal thought, including the Enneagram. We also discuss future research directions in this field.

## Tailoring meditation in ancient times

Interest in contemplative practices has grown inexorably over the last few decades (Garcia-Campayo et al., 2021).

According to various studies, the lifetime prevalence of meditation in Western countries, such as the United States, is 5.2%. Compared to non-meditators, the profile of meditators was more likely to be aged 40 to 64 years, female, non-Hispanic White, residing in the West, at least college-educated, not in a relationship, diagnosed with one or more chronic conditions, smokers, consumers of alcohol and physically active. Meditation was mainly used for general wellness (76.2%), improving energy (60.0%), and aiding memory or concentration (50.0%) (Cramer et al., 2016). Recent studies demonstrate that meditation practice extends to other population groups such as indigenous Americans, individuals ages 65 years and older, and individuals experiencing moderate or severe psychological distress (Davies et al., 2024). Similar findings have been reported in Australia (Lauche et al., 2019) and Iceland (Orlygsdottir et al., 2021).

One of the most challenging tasks for a meditation teacher is to match different forms of meditation with the personality types of meditation practitioners. Which meditative practices are more effective or useful for each individual? What kind of meditation do different people prefer? Is there any connection between meditation styles and personality traits, and which classification would be more reliable for this purpose? Finding answers to these questions is one of the greatest challenges in the field of contemplative practices.

Traditionally, masters of meditation in past centuries, who possessed deep insights into the mind of their disciples, would recommend the most suitable kind of meditation according to the specific characteristics of each individual. The permission and qualification to practice a particular tantric deity is called an ‘empowerment’, as it enables the recipient to engage in the practice of the generation and completion stages of that particular deity, ultimately leading to its accomplishment. The first ‘empowerment’ that one receives also constitutes one’s entrance to Vajrayana Buddhist path (Tulku, 1995). These complex processes, combined with the unwavering faith that disciples had in their masters, would enhance the effectiveness of meditation practice, regardless of type.

Most meditators in today’s modern societies no longer have a guru – a person invested with faith who advises practitioners on which specific form of meditation to follow. Instead, they practise on their own and choose for themselves the type of meditation that best suits their preferences and intuition. Even when such masters of meditation are available, the reality of our overcrowded world means that they have hundreds or even thousands of disciples, ruling out a close personal relationship and individual supervision in most cases. Rather than giving any specific advice on the best form of meditation for the particular individual, they will provide general teachings on certain practices. Only when gurus have a more limited number of followers can they devote the necessary time to tailor a meditation practice to their needs. In such cases, they endeavor to align the meditation techniques with the meditator based on advice from sacred texts while also relying on their intuition or wisdom. However, is this the best method, or could it be improved by modern personality psychology?

## Personality traits and meditation

Assessing personality is one of the most difficult tasks for mental health professionals, and inter-rater reliability tends to be low (Vernon, 2015). Currently, one of the most widely accepted assessment models for personality in psychiatry and psychology is the Big Five Personality Traits model, also known as OCEAN (Roccas et al., 2002), which stands for the five factors included in this theory: Openness to experience, Conscientiousness, Extraversion, Agreeableness and Neuroticism.

There are some research studies on the relationship between personality and meditation. A meta-analysis by Buric et al. (2022) studied the characteristics of meditators in relation to their response to meditation. It reported that higher levels of psychopathology or depression in subjects were associated with a decline in their mental health experienced after a meditation intervention. In contrast, interpersonal variables, motivation, medical conditions and mindfulness showed higher levels of positive meditation outcomes. Finally, demographics, self-concept, length of meditation practice and psychological traits – the object of this paper – did not significantly influence their response to meditation. The authors argue that their findings indicate that personality traits, as defined by frameworks such as the Big Five theory, do not significantly affect how participants respond to meditation. A similar conclusion was reached by Nyklicek and Irnischer (2017), who found no moderating effects of personality factors when controlling for baseline mood, with the exception of neuroticism, which was shown to be associated with delayed benefit. Tang and Braver (2020) suggest that Openness to Experience is the OCEAN trait most associated with positive outcomes and engagement in mindfulness meditation; however, there are no studies on specific meditation techniques. Another study on personality and type of meditation found that individuals with high neuroticism tend to benefit more from concentration and observing thoughts, while those with high extroversion obtain more positive responses from humming and walking meditation (Matko and Sedlmeier, 2023).”

Personality typologies can be found that are related to meditation. Several such classifications exist in Buddhism, such as: (a) personalities associated with the jhana practice and (b) the five Buddha families. The Enneagram is another personality classification associated with contemplation that has been used.

### Personalities associated with jhanas

Jhanas are states of one-pointed absorption characterized by a reduced awareness of one’s surroundings. The Pali Canon describes four progressive states known as *rūpa jhāna* (‘form *jhāna*’) and four additional meditative attainments that are referred to as *arūpa* (‘without form’) (Buddhaghosa, 1956). To attain these states, forty objects of meditation can be utilized, of which the chosen object should be appropriate to one’s character and temperament. Buddhist teachings mention a total of six character types, and a meditator may identify with any one of these types or a combination of them (Buddhaghosa, 1956).

It is essential to have a qualified and experienced meditation teacher, ideally a personal teacher who can be considered a good friend (*kalyanamitta*), to assist the meditator in choosing an appropriate meditation object. The teacher can explain the meditation process and guide the practitioner’s progress. Table 1 summarizes the recommended types of meditation for attaining jhana based on the meditator’s personality characteristics.

**Table 1:** Relationship between personality and the object of meditation to attain jhanas (Buddhaghosa, 1956).

Character	Recommended meditation
Lustful character (*lobha carita*): a person with strong feelings of sexual desire	Foulness of the body (it is a practice to break the illusion that the body is a permanent part of a permanent self. It brings attention to the temporary nature of the physical body)
Hateful character (*dosa carita*): an individual filled with hatred	The four brahmaviharas – loving-kindness, compassion, empathetic joy and equanimity – and the four color kasinas. Kasinas are basic visual objects of meditation. The four color kasinas are blue, yellow, red and white.
Deluded character (*moha carita*): a person believing something that is not true	Impermanence, or the fact that the existence of all objects and beings lasts for only a limited time. It is considered one of the main teachings of Buddhism
Faithful character (*saddha carita*): a character steadfast in affection or allegiance	The three jewels: Buddha, dharma or Buddhist teachings, and sangha or assembly of Buddhist practitioners. Or two qualities: morality (principles concerning the distinction between right and wrong) and generosity (the quality of being kind and generous)
Intellectual character (*panna carita*): a person possessing a highly developed intellect	Contemplation on death (meditation on the process of death and dying). Analysis of the four elements (the meditator sees the body as composed of the same material as everything else in nature: earth, water, fire and wind)
Speculative character (*vitakka carita*): an individual with a trend to conjecture	Mindful breathing (to focus our attention on our breath)

We can see a tailoring of meditation followed by thousands of meditators over the centuries.

### The five Buddha families

In Mahayana and Vajrayana Buddhism, the five Buddha families, frequently depicted in mandalas, are considered emanations or representations of the five qualities of the first Buddha, known as Adi-Buddha. This figure is not the historical Shakyamuni Buddha but represents the Dharmakaya, the unmanifested, inconceivable aspect of a Buddha out of which Buddhas arise and to which they return after their dissolution (Rockwell, 2002).

These qualities are represented by five couples, each comprising a masculine and a feminine Buddha; according to Buddhist mythology, they are associated with different directions (north, south, east, west or centre), colors (blue, yellow, red, green, or white), *mudrā* (the Sanskrit word for gesture) and symbols. Furthermore, they each embody a different aspect, type of evil and cosmic element, and they each have a spiritual son, as well as different animal vehicles (elephant, lion, peacock, garuda or dragon).

There are many more characteristics associated with each Buddha family, but for the purposes of this paper, we will focus only on the personality traits and identify them using the name of the masculine Buddha. The most common names for these Buddhas are Vairocana, Akshobhya, Ratnasambhava, Amithaba, and Amoghasiddhi. These five males Buddhas are also considered a classification of human psychological subtypes. All individuals, including meditators, are believed to belong to one of these categories, and a specific meditation should be tailored to each (Table 2).

**Table 2:** Shows the types of meditation suggested by Dhiravamsa (2011) based on the main ego fixation of each enneatype.

Enneatype	Main ego fixation	Meditation
Type 1. Perfectionist: self-controlled and rational	Resentment	Acceptance of imperfections and self-compassion
Type 2. Helper: generous and caring	Flattery	Loving-kindness and modesty
Type 3. Achiever: success-oriented and pragmatic	Vanity	Self-reflection on happiness as an inner experience
Type 4. Romantic: sensitive and dramatic	Melancholy	Gratitude for life
Type 5. Observer: cerebral and isolated	Stinginess	Mindfulness and sharing with others
Type 6. Loyalist: committed and responsible	Cowardice	Courage and Self-confidence
Type 7. Enthusiast: versatile and scattered	Planning	Sobriety and avoidance of compulsive searching of new experiences
Type 8. Challenger: dominating and self-confident	Vengeance	Surrendering and openness
Type 9. Peacemaker: easygoing and agreeable	Indolence	Mindful movement and action and presence

The Vairocana family is characterized by the delusion of ignorance and dullness, for which meditation on wisdom (the awareness of the true nature of reality, i.e., impermanence, suffering and emptiness) and non-duality (a way of living life, whereby one feels the interconnection with everything in each moment) is recommended for meditators who belong to it. The Akshobya family is primarily associated with the delusion of anger, so peace meditation is suggested for its members. Meditators whose main delusion is pride belong to Ratnasambava family and should practise equanimity (the quality that allows us to remain open and balanced in the midst of any changes). Individuals dominated by desirous attachment, the main trait of Amitabha family, benefit from meditation on impermanence. Finally, meditators characterized by jealousy, the mark of the Amoghasiddhi family, should focus on loving-kindness meditation.

While both Buddhist classifications – personalities associated with jhanas and the five Buddha families – are quite distinct, they have been in use for centuries without assessment by modern scientific methods.

### The Enneagram

The Enneagram is a model of human mind typologies described by Oscar Ichazo that draws on theories by different humanistic psychology authors. It describes nine interconnected personality types, referred to as enneatypes (Naranjo, 1995). While there are different schools of thought and authors who may not always agree on certain aspects, these nine types are generally accepted. The lack of scientific evidence supporting this classification may be due to limited research; however, its validity appears promising (Alexander and Schnipke, 2020). The Enneagram has been employed by therapists to enhance their understanding of object relations, prominent defences, core motivations and self-awareness.

There are currently no studies regarding the usefulness of this classification; however, the Enneagram has been associated with traditional Buddhist teachings (Piver, 2022), although Piver does not present an academic overview of correlations between the systems. Instead, her book offers a personal exploration of Buddhist teachings on liberation from suffering and their relationship to the Enneagram, illustrating through Buddhist teachings for each of the nine enneatypes that Enneagram is able to offer support for a compassionate and cognizant life, regardless of spiritual path (including the path of no path).

## Future suggestions

Tailoring meditation is a fascinating area of research that deserves further exploration. While some tentative proposals have been made, there is currently no solid evidence regarding its effectiveness. Two of the most significant attempts to tailor personality to mediation type are the work by Jagielski et al. (2020), a health-related study on women with breast cancer, and the work by Nyklicek and Irnischer (2017), an analysis of the effect of personality on the outcomes of meditation in the workplace.

We have identified three classifications of personality based on different traits, each one emphasizing distinct aspects of human psychism. Given the difficulty of comparison, they should be assessed independently. One of the challenges is developing reliable questionnaires to measure the different personality subtypes outlined in the three taxonomies. Another is accurately defining the meditations to be assessed. Finally, there must be agreement on what constitutes improvement in meditation. An important limitation of this paper is that we focus only on participant characteristics as sources of variability in responses to meditation, although we acknowledge that contextual factors, such as characteristics of the meditation teacher or group processes, can also play a role. Our aim has been to review the current state of research in this area and reflect on future directions to advance this field.

## Data Availability

The raw data supporting the conclusions of this article will be made available by the authors, without undue reservation.
